# Susceptibility of Broiler Chickens to Coccidiosis When Fed Subclinical Doses of Deoxynivalenol and Fumonisins—Special Emphasis on the Immunological Response and the Mycotoxin Interaction

**DOI:** 10.3390/toxins8080231

**Published:** 2016-07-27

**Authors:** Bertrand Grenier, Ilse Dohnal, Revathi Shanmugasundaram, Susan D. Eicher, Ramesh K. Selvaraj, Gerd Schatzmayr, Todd J. Applegate

**Affiliations:** 1 Department of Animal Sciences, Purdue University, W. Lafayette, IN 47907, USA; bertrand.grenier@biomin.net; 2 BIOMIN Research Center, Tulln 3430, Austria; ilse.dohnal@biomin.net (I.D.); gerd.schatzmayr@biomin.net (G.S.); 3 Department of Animal Sciences, Ohio Agricultural Research and Development Center, Wooster, OH 44691, USA; shanmugasundaram.2@osu.edu (R.S.); selvaraj.7@osu.edu (R.K.S.); 4 Livestock Behavior Research Unit, Agricultural Research Service, USDA, W. Lafayette, IN 47907, USA; Susan.Eicher@ars.usda.gov; 5 Department of Poultry Science, University of Georgia, Athens, GA 30602, USA

**Keywords:** mycotoxins, coccidiosis, challenge, interaction, intestinal immune response

## Abstract

Deoxynivalenol (DON) and fumonisins (FB) are the most frequently encountered mycotoxins produced by *Fusarium* species in livestock diets. The effect of subclinical doses of mycotoxins in chickens is largely unknown, and in particular the susceptibility of birds to pathogenic challenge when fed these fungal metabolites. Therefore, the present study reports the effects of DON and FB on chickens challenged with *Eimeria spp*, responsible for coccidiosis. Broilers were fed diets from hatch to day 20, containing no mycotoxins, 1.5 mg DON/kg, 20 mg FB/kg, or both toxins (12 pens/diet; 7 birds/pen). At day 14, six pens of birds per diet (half of the birds) were challenged with a 25×-recommended dose of coccidial vaccine, and all birds (challenged and unchallenged) were sampled 6 days later. As expected, performance of birds was strongly affected by the coccidial challenge. Ingestion of mycotoxins did not further affect the growth but repartitioned the rate of reduction (between the fraction due to the change in maintenance and feed efficiency), and reduced apparent nitrogen digestibility. Intestinal lesions and number of oocysts in the jejunal mucosa and feces of challenged birds were more frequent and intense in the birds fed mycotoxins than in birds fed control feed. The upregulation of cytokines (interleukin (IL) IL-1β, IL-6, IL-8 and IL-10) following coccidial infection was higher in the jejunum of birds fed mycotoxins. Further, the higher intestinal immune response was associated with a higher percentage of T lymphocytes CD4^+^CD25^+^, also called T_regs_, observed in the cecal tonsils of challenged birds fed mycotoxins. Interestingly, the increase in FB biomarker of exposure (sphinganine/sphingosine ratio in serum and liver) suggested a higher absorption and bioavailability of FB in challenged birds. The interaction of DON and FB was very dependent on the endpoint assessed, with three endpoints reporting antagonism, nine additivity, and two synergism. In conclusion, subclinical doses of DON and FB showed little effects in unchallenged chickens, but seem to result in metabolic and immunologic disturbances that amplify the severity of coccidiosis.

## 1. Introduction

Mycotoxins are structurally diverse low-molecular weight metabolites produced by various molds belonging chiefly to the *Aspergillus*, *Penicillium,* or *Fusarium* species. Effects in animals following the ingestion of these fungal compounds vary from acute, overt disease with high morbidity and death to chronic, decreased resistance to pathogens and reduced animal productivity [1,2]. However, the major problem associated with animal feed contaminated with mycotoxins is not acute disease episodes, but rather the ingestion of low level of toxins which may cause an array of metabolic, physiologic, and immunologic disturbances [3].

Deoxynivalenol (DON) and fumonisins (FB), both from *Fusarium* species, are the most frequent mycotoxins found in feed and feedstuffs [4]. Poultry have long-been considered relatively resistant to mycotoxins, especially to toxins from *Fusarium*. However, very recent investigations have shown that concentrations of mycotoxins below the EU and US limits or at concentrations lower than those that would cause overt clinical mycotoxicosis, may significantly affect the performance and health of birds [5,6,7,8,9,10]. Many reasons may account for these new findings. First, feeding trials conducted prior to the 1990s have used different strains of birds, and it has been noted a differential sensitivity to mycotoxins, especially aflatoxins, between traditional and modern poultry production [11]. This might be a consequence of genetic selection for rapid growth which may have altered metabolic and nutrient partitioning, and has long-been criticized in reducing immunity [12]. Second, investigations are now growing in the field of gut health, and recent reports on mycotoxins have primarily focused on their effects in the gastro-intestinal tract (GIT). In poultry, DON and FB are very poorly absorbed (approximately 10% and 1%, respectively), inferring that a substantial part of mycotoxins remains in the GIT and expose the intestinal cells to high concentrations of toxins. This might result in the impairment of intestinal functions, and also favor the growth and colonization of digestive pathogens in poultry. For instance, Antonissen et al. [8] clearly demonstrated that DON predisposes birds to necrotic enteritis by providing the necessary growth substrate for proliferation of *Clostridium perfringens*, due to the DON effect on leakage of plasma amino acids into the intestinal lumen. 

Another predisposing factor to necrotic enteritis is mucosal damage caused by coccidial pathogens. Avian coccidiosis is a disease of birds caused by protozoal parasites of the genus *Eimeria* [13]. Coccidiosis is still one of the most costly diseases in modern broiler production and associated with global economic losses of $3 billion annually as a result of lost productivity and control measures [14]. As a result of drug resistance by *Eimeria* species, interest has increased in the use of live vaccines for disease control. They are typically administered to chicks during the first 1 to 7 days of life but it requires at least 7 to 10 days for the stimulation of the acquired immune response [15,16]. Consequently, chickens are most susceptible to pathogens during the first weeks post hatch. The response of birds to coccidiosis when fed mycotoxins has been already investigated by Girgis et al. [5,17,18], wherein chickens following challenge had a delayed immune response when given grains naturally contaminated with multiple *Fusarium* mycotoxins (DON, 15-acetyl DON, and zearalenone).

The aim of the present study was not only to provide extra data on the effects of mycotoxins during a coccidiosis outbreak, but also to determine the interaction between the mycotoxins DON and FB in chickens. As previously mentioned, DON and FB co-occur in feed and feedstuffs, and this is not an exception but a common situation with co-occurrence of mycotoxins reaching up to 50% in recent surveys [4]. This supports the real need to assess their interactions in animals and to conclude whether the combination of two toxins lead to antagonistic, additive, or synergistic effects [19]. To this end, mathematical models such as isobolograms are very useful to conclude the type of effects resulting from the combination of different concentrations of toxins/drugs [20]. Very recently, this model has been applied and studied on intestinal epithelial cells treated with a binary or ternary mixture of different mycotoxins, belonging to the trichothecene group [21]. The major highlight of this study concerned the different outcome when applying a mixture of low or high doses to cells. Although such models provide substantial information, the use of a gradient concentration of toxins in combination is not really conceivable in animal experiments, or only limited to performance analysis. Besides, cell viability is very often the only parameter examined in these in vitro interaction studies, but the endpoint assessed has been also reported as a critical factor in the outcome of interactions [19].

Therefore, we investigated the interaction of subclinical doses of DON and FB, alone or in combination in chickens with or without a challenge with *Eimeria* strains. To induce gut inflammation and mild lesions, we challenged birds with an overdose of coccidial vaccine, as previously reported [22]. The type of interaction between these two mycotoxins was determined by applying a two-way factorial ANOVA for each endpoint analyzed. Furthermore, to further investigate the intestinal immune response to *Eimeria spp*, a separate experiment was conducted with only DON, with particular focus on the cell-mediated response by analyzing the T cell population (CD4^+^ and CD8^+^ cells), especially the T_reg_ response (CD4^+^CD25^+^ cells, [23]) in the cecal tonsils.

## 2. Results

### 2.1. Experiment 1: Feeding Trial with Diets Contaminated with DON and FB, Alone or in Combination

#### 2.1.1. Performance, Clinical Signs, Lesions, and Oocyst Identification

##### Performance

**Figure 1:**
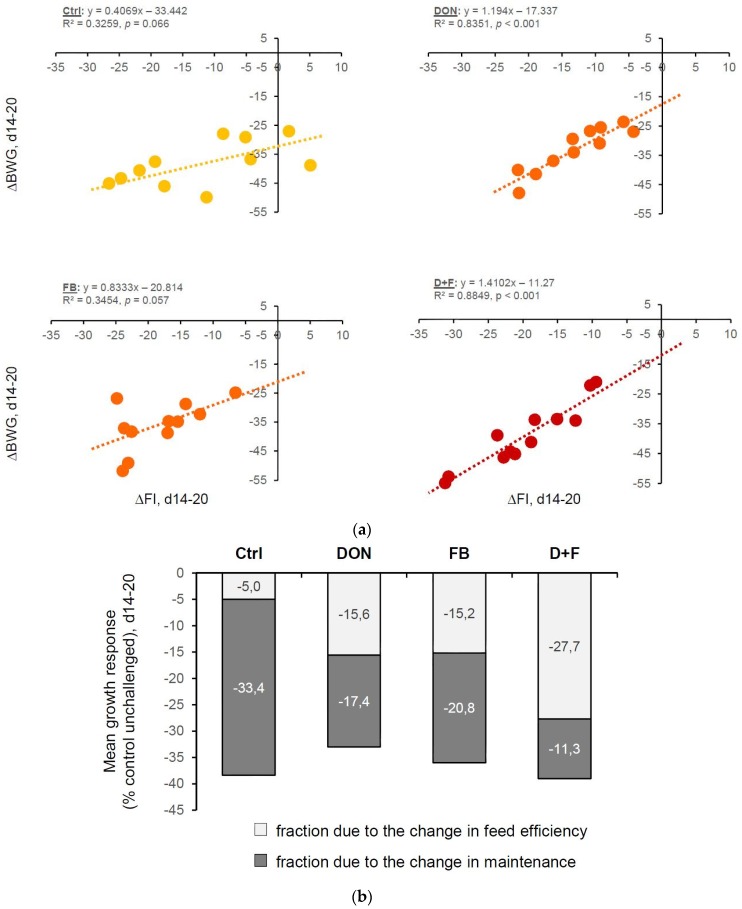
Relationship between the reductions in feed intake and growth rate (Experiment 1, based on Pastorelli et al. [24]): (**a**) Relationship between the change in growth (∆BWG, Body Weight Gain day 14–20) and feed intake (∆FI, Feed Intake day 14–20) of chickens challenged with a coccidial vaccine. Responses are expressed as results of the challenged chickens relative to that of unchallenged chickens on control feed (in %, 11 birds/diet). The lines represent the linear model adjustments; (**b**) Partitioning of the reduction in the average growth rate following the challenge between the fraction due to change in maintenance requirement (i.e., not associated with a reduction in feed intake) or due to the change in feed efficiency (i.e., associated with a reduction in feed intake).

No significant changes in the body weight gain and the feed intake were observed before the coccidial challenge or in the unchallenged birds (Table 1). By contrast, the body weight gain (day 14–20) of the birds challenged with the vaccine overdose was dramatically reduced by 38.4, 33.0, 36.0, and 39.0% for control, DON, FB, and DON + FB, respectively, compared to the unchallenged control birds (Table 1, Figure 1). Although there was no significant difference between the diets, the relationship between the change in growth and feed intake of birds challenged with the coccidial vaccine was different between diets (Figure 1). The analysis, based on Pastorelli et al. [24], showed that the changes observed in the growth of control birds were mostly due to the change in maintenance. By contrast, the partitioning was different in birds fed contaminated diets (for a similar reduced body weight gain), with the fraction due to the change in feed efficiency increasing according to the presence of mycotoxins in the diets. To determine whether mycotoxins have a prolonged impact on the performance following coccidiosis outbreak, extra birds were kept for two weeks (recovery phase) and the BWG and FI were evaluated. As shown in Table 1, the challenged birds were all able to perform as good as unchallenged control birds two weeks post-challenge. No significant differences in the recovery (i.e., on toxin-free diet from 20 to 34 days of age) were observed.

##### Clinical Signs

No mortality due to the diets or the coccidial vaccine was observed over the experimental phase. Following the challenge, blood was observed in the droppings but was not attributable to an experimental diet. In addition, excreta of these birds appeared very dry suggesting issues in water intake and/or absorption.

##### Gross Lesions

**Figure 2:**
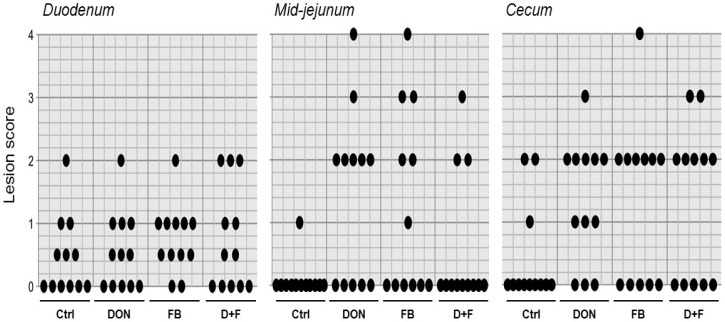
Lesion scoring in the gastrointestinal tract (GIT) of chickens challenged with the coccidical vaccine (Experiment 1). Each plot reports the score of individual data of 12 birds per diet. No significant differences between contaminated diets and control diet were observed in the duodenum. In the mid-jejunum, significant differences were noted in the mean lesion score (±SEM): 1.42 ± 0.40 (*p* = 0.029), 1.25 ± 0.43 (*p* = 0.084), and 0.58 ± 0.31 (*p* = 0.442), respectively, for DON, FB and DON + FB compared to control diet, 0.08 ± 0.08. Similarly, the mean lesion scores significantly differed in the ceca of birds: 1.33 ± 0.28 (*p* = 0.019), 1.33 ± 0.38 (*p* = 0.049), and 1.33 ± 0.36 (*p* = 0.041), respectively, for DON, FB and DON + FB compared to control diet, 0.42 ± 0.23.

Typical lesions reported in outbreaks of chicken coccidiosis were observed in the present experiment. In the upper part of the GIT, the lesions observed were attributed to *Eimeria acervulina* and *mivati* contained in the vaccine. However, the intensity of the lesions was rather mild, and no difference was observed between diets (Figure 2). By contrast, in the jejunum of birds fed contaminated diets, the intestinal tissue exhibited moderate lesions with presence of blood in a few birds (scored 3 and 4). This effect was most likely associated with *Eimeria maxima*, and the pathology was more pronounced in presence of mycotoxins (Figure 2; mean lesion score (±SEM): 1.42 ± 0.40 (*p* = 0.029), 1.25 ± 0.43 (*p* = 0.084), and 0.58 ± 0.31 (*p* = 0.442), respectively, for DON, FB and DON + FB compared to control diet, 0.08 ± 0.08). *Eimeria tenella* targets specifically the chicken’s ceca, and again, the lesions observed in the ceca in the present study were assumedly attributed to this strain. Bleeding in ceca was observed very frequently, especially in the birds fed mycotoxins (Figure 2), and was even more severe in some birds (scored 3 and 4) along with an absence of normal ceca contents. The occurrence and intensity of lesions were significantly more pronounced in the birds fed the contaminated diets (mean lesion score (±SEM): 1.33 ± 0.28 (*p* = 0.019), 1.33 ± 0.38 (*p* = 0.049), and 1.33 ± 0.36 (*p* = 0.041), respectively, for DON, FB and DON + FB compared to control diet, 0.42 ± 0.23).

##### Oocyst Identification

**Figure 3:**
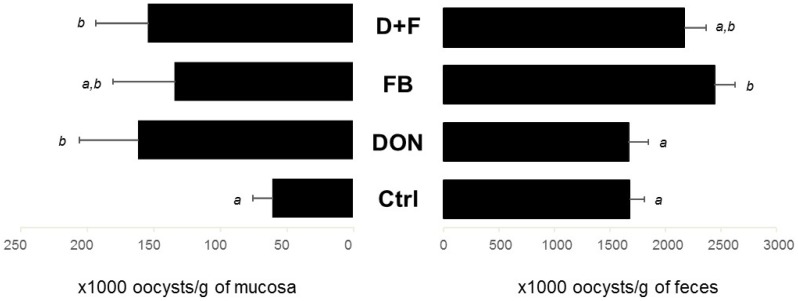
Oocyst counting in the jejunal mucosa and excreta of chickens challenged with the coccidial vaccine (Experiment 1). McMaster slide was used to count the floating oocysts under microscope at 10× magnification. Values are mean ± SEM for six birds (mucosa) and six pools (excreta). Means with no common superscript are significantly different (*p <* 0.05).

The oocyst number was estimated in both jejunal mucosa and excreta 6 d-post challenge. As mentioned in the Materials and Methods, due to differences in size among *Eimeria* strains in the vaccine, only visible oocysts were counted and the results are mostly based on the counting of *E. maxima* and *tenella*, bigger than *E.* *acervulina* and *mivati*. Nonetheless, the counting in the jejunum is considered accurate as *E. maxima* specifically target the mid-intestine. The analysis in the jejunum showed that the ingestion of mycotoxins resulted in a higher number of oocysts in the intestinal tissue compared to the birds on control feed (Figure 3; *p* = 0.049 for DON, *p* = 0.192 for FB, and *p* = 0.043 for DON + FB, in comparison to the control group). An increase was also observed in the excreta of birds fed FB and DON + FB compared to control birds (Figure 3; *p* = 0.027 and 0.221, respectively). Given all of the birds were reared in the same room, excreta from unchallenged birds were collected, pooled per diet, and subjected to the McMaster method. None of the excreta analyzed displayed the presence of oocysts, ruling out a possible cross-contamination between challenged and unchallenged birds.

#### 2.1.2. Gut Integrity

##### Villi Height

The villi in the jejunum of unchallenged birds were not significantly different regardless of the feed ingested (Table 2). No data are available regarding the villi height in the intestine of challenged birds as those birds exhibited a discontinuous epithelium with severe degradation of the villi. This has already been reported elsewhere [17].

##### Apparent digestibility

The ingestion of DON and FB, alone or in combination had no effect on the apparent DM and N digestibility of unchallenged birds (Table 2). As expected, the challenge of the birds with the coccidial vaccine strongly impaired the apparent digestibility measured in the ileum. A further decrease was observed in the group treated with DON + FB on the apparent N digestibility (Table 2; *p* = 0.031).

##### Fumonisin Exposure

**Figure 4:**
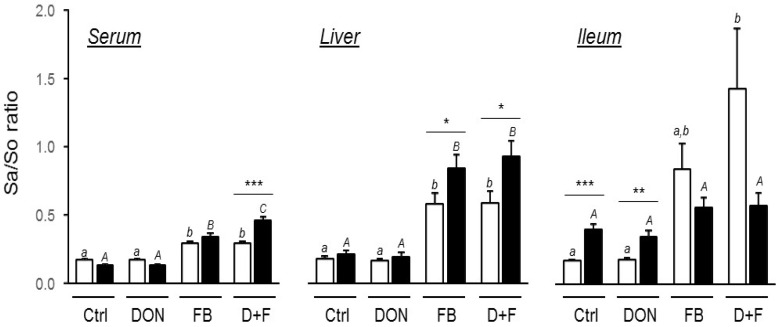
Biomarker of exposure to FB, sphinganine/sphingosine ratio in the serum, liver and ileum of unchallenged and challenged chickens (Experiment 1). White and black bars represent unchallenged and challenged birds, respectively. Values are mean ± SEM for six birds. *, *p* ≤ 0.05; ***, *p* ≤ 0.001. Means with no common superscript (lower and upper case for unchallenged and challenged birds, respectively) are significantly different (*p <* 0.05).

The use of the Sa/So ratio as a biomarker of exposure to FB is very well accepted [25]. As expected, the ingestion of FB either alone or in combination with DON significantly increased the Sa/So ratio in both unchallenged and challenged birds in comparison to other diets (Figure 4). Interestingly, this ratio is significantly higher in the serum and liver of birds challenged with the vaccine than birds not challenged (Figure 4; +59%, *p* < 0.001 in the serum of DON + FB group; +47% and 58%, *p* = 0.045 and 0.037 in the liver of FB and DON + FB, respectively). Conversely, the ratio in the ileum of challenged birds fed FB and DON + FB was very low compared to unchallenged birds fed the same diets (Figure 4; −33% and −59%, *p* = 0.193 and 0.090 in the ileum of FB and DON + FB, respectively). In addition, it seems that the challenge had an effect on the basal level of sphingoid bases in the small intestine since both control and DON diets displayed higher ratios than unchallenged birds fed the same diets. The individual values for Sa and So showed a considerable increase of both sphingoid bases in the challenged birds regardless of the diets (data not shown).

##### Gene Expression

**Figure 5:**
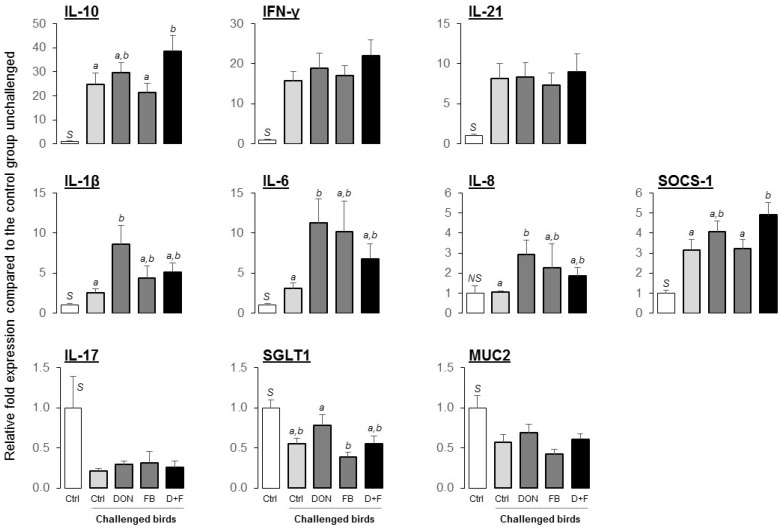
Gene expression in the jejunum of chickens challenged with coccidial vaccine (Experiment 1). The white bar (first bar in each plot) represents the mean value of the unchallenged birds on control feed. The darker bars represent the mean value of the challenged birds on experimental diets, and expressed relative to the unchallenged control group. Values are mean ± SEM for six birds. Means with no common superscript are significantly different (*p <* 0.05). S, Significant and NS, Non-Significant difference between both control groups, unchallenged and challenged.

The mRNA levels of SGLT1 (sodium glucose co-transporter 1) and MUC2 (mucin 2) were determined in the jejunum of birds and both were found significantly decreased following the coccidial challenge (Figure 5). However, there were no significant differences between the contaminated diets and the control diet.

#### 2.1.3. Gut Inflammation

##### Gene Expression

Coccidiosis is well known to result in strong intestinal inflammation that is partly mediated by an overexpression of cytokines [26]. As expected, some cytokines were strongly up-regulated after challenging birds with the coccidial vaccine, especially IL-10, IFN-γ, IL-21 and IL-6 (Figure 5). Surprisingly, expression of IL-17 was found to be significantly decreased after the challenge, IL-17 usually being a cytokine reported to increase during intestinal inflammation. The response to the challenge in presence of mycotoxins was clearly more pronounced, especially the IL-1β, IL-6, and IL-8 responses in the jejunum of birds fed DON alone (Figure 5; 3.3, 3.7 and 2.8 fold higher than in challenged birds on control feed, respectively). On the other hand, IL-10 and SOCS-1, two cytokines involved in the regulation of inflammation, were only up-regulated when both mycotoxins were combined (*p* = 0.044 and 0.046, respectively).

#### 2.1.4. Interaction between Mycotoxins

##### Factorial ANOVA

To determine the interaction between mycotoxins, the model of two-way factorial ANOVA was applied. Statistical output with *p* value > 0.05 was considered as an additive interaction, and *p* value < 0.05 was considered as either a synergistic or antagonistic interaction. The outcome of the statistical analysis for challenged birds is reported in Table 3. In short, 20 different endpoints were assessed in the present study, and additive effect of DON and FB was noted in nine endpoints, synergistic effect in two endpoints, antagonistic effect in three endpoints, and no difference with the challenged control group in six endpoints.

### 2.2. Experiment 2: Feeding Trial with Diets Contaminated with DON

#### 2.2.1. Gut Inflammation

##### Lymphocytes T Cells

The population of CD4^+^CD25^+^ lymphocytes corresponding to the T_reg_ lymphocytes in chickens was low in the cecal tonsils of unchallenged birds regardless of diet (Table 4). By contrast, the coccidial challenge induced an augmentation of this lymphocyte subset in the cecal tonsils, with a further increase when birds fed DON (Table 4; +275%, *p* = 0.005 and +67%, *p* = 0.041 in comparison to control unchallenged and challenged, respectively). The CD8^+^ population was not affected by DON ingestion, but a significant difference was seen between unchallenged and challenged birds on basal feed.

##### Gene Expression

The coccidial challenge significantly increased the mRNA levels of IL-6, IFN-γ and IL-10 in the cecal tonsils of birds, and ingestion of DON resulted in higher expression of IFN-γ as well as IL-1β (*p* = 0.029 and 0.137, respectively, compared to challenged control birds, Table 4).

## 3. Discussion

Prior to the 2000s, many trials were conducted in poultry, especially in chickens, to investigate the effects of mycotoxins. This has resulted in substantial and valuable information regarding the toxicity of these fungal metabolites, and enabled the establishment of recommended thresholds set by governmental authorities. Among mycotoxins, aflatoxins and ochratoxins have been considered the most harmful toxins in poultry [27]. In contrast to swine, mycotoxins produced by *Fusarium* species, such as deoxynivalenol (DON) and fumonisins (FB), have not been considered as a major threat in poultry production, as more than 15 mg DON/kg and 80–100 mg FB/kg of diet are required to reduce performance in chickens [28,29,30]. However, as pointed out by Yunus et al. [31], recent reports showed temporary reduction in broiler performance exposed to lower doses of DON (also shown for aflatoxins). The modern broiler has a faster growth rate that can affect the relative sensitivity of birds to mycotoxins in comparison to traditional broiler prior to the 2000s. This is in agreement with very recent reports showing the toxic effect of DON in chickens at doses below the recommended limits and close to field conditions (1 to 5 mg/kg; as reviewed by Murugesan et al. [27]). Taken together, there is a need to reevaluate the effects of lower doses of mycotoxins, especially subclinical doses that do not induce clinical signs but may predispose birds to metabolic and immunologic disorders.

In the present study, the concentrations of DON and FB used did not affect the performance and health of birds raised in healthy conditions. However, under conditions of pathogenic challenge, both mycotoxins seemed to impair the response of chickens to coccidiosis. The activation of the immune defense following an antigenic challenge makes the animals more susceptible to mycotoxins (reviewed in Oswald et al. [1]). This is attributed to their mechanisms of toxicity. DON inhibits the synthesis of proteins through the binding to eukaryotic ribosomes [32], and FB disrupt the sphingolipid metabolism through the inhibition of ceramide synthase [25]. Although the cellular effects are very different, both toxins act on active and dynamic processes inside the cell, and cells exhibiting high protein activity and/or fast renewal of sphingolipid membrane, such as lymphocytes and intestinal epithelial cells facing antigenic challenge, become target of these mycotoxins. Additionally, both fusariotoxins are known to be poorly absorbed in the GIT of poultry, inferring they remain in the intestinal lumen, interacting with intestinal cells but also with digestive pathogens.

DON and FB, alone or in combination, did not result in further reduction of growth of challenged birds (administration of coccidial vaccine in control birds resulted in −38% of growth compared to unchallenged birds within a week). Interestingly, when applying the model of Pastorelli et al. [24], it is quite obvious that the changes in the growth of challenged birds on control feed are due to high maintenance requirements (diversion of feed to support the higher requirement of the immune system in nutrients). By contrast, it seemed that there was a repartitioning of the reduction in the average growth rate following challenge with *Eimeria spp* and mycotoxins, between the fraction due to the change in maintenance requirement (i.e., not associated with a reduction in feed intake) or due to the change in feed efficiency (i.e., associated with a reduction in feed intake). The fraction due to change in feed efficiency was almost two-fold higher in the co-contaminated diet than in the individual ones. This is in agreement with Pastorelli et al. [24], showing the same partitioning during mycotoxicosis. This also suggests that the reduction in feed intake (seen especially in DON + FB group from 14 to 20 d) resulted in less absorption of nutrients, and therefore less energy to properly respond and clear the intestinal parasites. This might partly explain our results on the higher number of oocysts or lesions or cytokine expression. The recovery phase included in the experimental design was intended to find out whether birds fed mycotoxins during the first phase would perform as good as birds on control feed following the coccidial challenge. No changes between treatments were observed in terms of body weight gain and feed intake during the recovery phase. However, all of the birds were fed uncontaminated feed during this recovery phase and a different outcome might have occurred if birds were kept on their respective diets, such as reported in Girgis et al. [5] (effect on the restoration of the CD8^+^ population).

Typical lesions of the *Eimeria* strains used were observed in the GIT, especially in the cecum of birds where presence of hemorrhages most likely account for blood in the droppings, regardless of the diets. Surprisingly, the challenged birds on control feed did not exhibit any gross lesions specific of *Eimeria maxima* in the jejunum. It is possible that the concentration of *E. maxima* sporulated oocysts in the 25×-vaccine was not high enough or virulent enough to induce apparent lesions. By contrast, the birds fed mycotoxins had lesions typical of *E. maxima*, such as petechiae, orange mucus, and for some, blood clots. This might also explain the results of oocyst counting in the mucosa, with fewer numbers of oocysts in the jejunum of birds on control feed than on contaminated feed. In birds fed mycotoxins, this may imply a lower ability to clear *Eimeria spp* from the jejunum (prolonged invasion) or a higher invasion in the intestinal epithelium (increased development and multiplication of the parasites). In line with that, several up-regulations of cytokines related to an inflammatory response were seen in that section of the GIT of birds fed mycotoxins. The cecum of challenged birds fed mycotoxins was also very affected, with lesions significantly more intense than in the control birds. The effect of *E. tenella* in the cecum was obvious with many birds exhibiting large amount of blood in their ceca. As already mentioned, the number of oocysts was higher in the jejunum of birds fed diets containing DON and DON + FB, but also in the excreta of birds fed FB, and to a lesser extent DON + FB. This might have important implications in modern animal production that frequently involves the raising of large numbers of birds at high stocking densities. This provides an ideal opportunity for parasite transmission such as *Eimeria* that have an oral/fecal life cycle [13]. In addition, broiler producers routinely recycle or top dress litter for subsequent flocks because of high cost of bedding material [33].

A noteworthy finding in the present study is the differential increase of Sa/So ratios between unchallenged and challenged birds. The coccidial challenge seemed to increase the passage of FB through the intestinal barrier as the Sa/So ratios were significantly higher in both the serum and liver in comparison to unchallenged birds. The Sa/So ratio is very indicative of FB exposure, regardless of species, and dose–response has been already demonstrated in chickens [9,25,28]. As mentioned in the Results section, the measurement of villus height in the jejunum of challenged birds was not feasible as a result of tissue damage by the parasite development and replication. It is then reasonable to postulate that the oral challenge has strongly compromised the barrier function of the intestine, and thereby increased the bioavailability of FB. No conclusions can be drawn regarding DON bioavailability but the question should be addressed too. The increase of both sphingoid bases Sa and So concentrations, regardless of the diets, in the ileum of challenged birds also attested of the condition of the intestinal tissue within the period of coccidial infection. As a consequence of this release of sphingolipids from the intestinal cell membranes by *Eimeria spp*, the Sa/So ratios were not significantly different between the diets. This deterioration of the gut epithelium is also most likely responsible for the reduced digestibility of nutrients observed in challenged birds, as well as the decreased expression of SGLT1 which mediates the entry of glucose into the cell. This effect on nutrient transporters has already been described during infection with *E. maxima* [34]. In unchallenged birds, our results are in agreement with Antonissen et al. [10] that also showed that the Sa/So ratios in plasma of birds fed FB alone or in combination with DON were the same. Indeed, it was hypothesized that the known increase of paracellular permeability by DON (through its effects on tight junctions) could at the same time increase the passage of other toxics, such as FB. Although these findings are based on Sa/So analysis, the same group also revealed the same year that DON has no influence on the oral bioavailability of FB_1_ in broilers by determining the FB concentration [35].

Another effect of coccidiosis, particularly well-studied by Lillehoj’s group [26,36,37], concerns the strong up-regulation of cytokines involved in inflammation and immune defense of the birds. The gene expression analysis in the present study has been performed in the jejunum of birds, and as previously mentioned, it is reasonable to ascribe our findings to the effects of *E. maxima*. Our results are in agreement with Hong et al. [37] who have challenged broilers with a similar concentration of *E. maxima* (10,000 sporulated oocysts). They first observed a dramatic elevation of cytokine expression in the very first days post-challenge (from 60 to 1600 fold for IL-6, IL-1β, IL-8, IL-17, and IFN-γ), and although their expressions were decreasing over time, at d 7 post challenge, these cytokines were still up-regulated and within the same range of what was observed in the present study, except for IL-17. This finding on the repressed expression of IL-17 was not expected, but was also confirmed in Experiment 2 regardless of the diets. Expression of IL-10, SOCS1 and IFN-γ were high in both experiments, and IL-10 and SOCS1 are produced by lymphocytes to control inflammation, such as T_regs_. Similarly, IFN-γ is well-known to mediate the resistance to different parasites [26]. Taken together, it might be speculated that these cytokines were helping the birds attenuate cytokine signaling and inflammation by negatively regulating the Th17 cells producing IL-17. Nonetheless, there was no further effect when birds were exposed to mycotoxins. By contrast, association of DON and FB resulted in higher levels of mRNA encoding for IL-10 and SOCS1, whereas IL-1β, IL-6 and IL-8 belonging to pro-inflammatory cytokines were further increased only after ingestion of DON alone (3 fold higher than challenged birds on control feed). Another gene indirectly related to inflammation is MUC2, the most abundant mucin in the intestine. The low expression of MUC2 observed in the present study is believed to also contribute to inflammation as its deficiency has been reported to increase bacterial translocation and inflammation [38]. In line with MUC2 expression, the very dry aspect of feces suggested that the mucus was severely compromised and could not play its role as a layer of protection in the intestine. However, all of the diets resulted in the same reduction of mRNA levels of MUC2. Cell-mediated immune responses are known to be predominant in the intestine of *Eimeria*-infected animals [26]. CD4^+^ cells, macrophages and intraepithelial lymphocytes are the main cells responsible for controlling primary infections, while CD8^+^ cells play the major role in immunity to secondary infections [26]. However, nothing is known about the subset CD4^+^CD25^+^ when birds are challenged with *Eimeria spp*. It has been recently demonstrated in chicken that CD4^+^CD25^+^ cells have T_reg_ properties (high IL-10, TGF-β amounts), and are present in high numbers in mucosal regions like cecal tonsils [23,39]. Therefore, the objective of the Experiment 2 was to provide new data in chickens and investigate the cellular response to coccidiosis with chickens fed higher concentration of DON. In the cecal tonsils, DON significantly increased the total number of CD4^+^ population following the challenge (in agreement with Girgis et al. [18]), and resulted in a dramatic increase in the CD4^+^CD25^+^ subset in comparison to unchallenged birds and challenged birds on control feed. In addition, the expression of IL-1β and IFN-γ was higher in the cecal tonsils of birds challenged and fed with DON than with control feed. Taken together, these results suggest that inflammation induced by the coccidial challenge was probably stronger in birds fed DON, and thereby these birds were requiring more T_regs_ to control inflammation. 

The present study also aimed to determine the type of interaction resulting from the combination of DON and FB. As already mentioned, co-occurrence of mycotoxins in feeds is not an exception but a norm. The data on toxicity of combined mycotoxins are still limited, especially on the effect of realistic doses [19]. To the best of our knowledge, the interaction of DON and FB was assessed in chickens in three studies. The first, previously mentioned, investigated the bioavailability of FB in presence of DON [35], and the second and the third studied the toxicological interaction of DON and FB [10,40]. However, one of the studies used very high contaminated feed, 15 mg DON/kg and 300 mg FB/kg [40], and it is unlikely to find these concentrations in naturally contaminated grains. The other study used slightly higher concentrations of DON and FB than the present work (4.5 and 24 mg/kg, respectively; [10]). In the present study, the doses used are considered low for DON (representative of field conditions) and moderate for FB (during unfavorable weather conditions) [3]. However, both were below the limits set by EU and US authorities and considered as subclinical doses in poultry. As a matter of fact, low but significant contamination of FB was also found in the control diet. This could be a reason behind the lack of dose–response regarding gene expression, as already demonstrated elsewhere [9]. However, in combination with DON, this led to more pronounced effects. As summarized in Table 3, the ingestion of both mycotoxins at the same time resulted in different type of interactions depending on the endpoints assessed. The endpoint assessed is crucial when investigating the interaction of mycotoxins or toxic compounds in general. Antonissen et al. [10] also showed different outcome when studying DON and FB in combination, with for instance antagonistic interaction on the expression of some intestinal nutrient transporters, but also synergism on the digestibility of methionine. Other important factors that can influence the outcome of an interaction are the doses used, the animal species, the age as well as the gender [19]. The in vivo toxicity of mycotoxin interaction cannot be predicted in vitro, and more feeding trials using mixture of toxins are needed to conclude on the effect of mycotoxin interaction. Even mycotoxins with similar modes of action may result in antagonistic effects.

## 4. Conclusions

In conclusion, chronic exposure to mycotoxins at concentrations that are not considered hazardous may result in metabolic and immunological disturbances in chickens and contribute to the severity and outbreak of pathologies, such as in coccidiosis.

## 5. Materials and Methods

### 5.1. Experiment 1: Feeding Trial with Diets Contaminated with DON and FB, Alone or in Combination

#### 5.1.1. Experimental Birds, Housing, Diet Formulation and Sampling

##### Diet Formulation

All animal care and use procedures for the experiment were approved by the Purdue University Animal Care and Use Committee (Protocol 1112000426; Approval 23rd of October 2012 for 3 years). The feeding trial was divided in two experimental phases: (i) d0–20, starter feed with birds fed experimental diets; and (ii) d21–34, grower feed with birds fed toxin-free diets. This 34-d feeding study was conducted with 1-d-old male broilers (Ross 708). Regarding the starter feed, four different experimental diets were prepared, one control diet without mycotoxin incorporation, and three diets containing FUM and DON alone and in combination. Two strains of *Fusarium*, *F. graminearum* DSM-4528 and *F. verticillioides* M-3125 were used to produce DON and FB, respectively. These strains were separately grown on rice. FB was produced as previously described [41], and DON was produced by Romer Labs (Tulln, Austria) in accordance to the protocol described by Altpeter et al. [42]. The homogenized extracts contained 26.0 and 5.7 g/kg DON and FB, respectively. These extracts were mixed with a small portion of the basal diet, and remixed with appropriate amount of basal feed to create the experimental diets. Diets were then remixed with 0.5% chromic oxide, which was fed from 16 to 20 days of age, to determine apparent nutrient digestibility. During the first phase (d0–20), experimental diets were formulated to contain 2 and/or 20 mg/kg of DON and/or FB, respectively. Basal grower feed was used during the second phase (day 21–34) without incorporation of mycotoxins. HPLC-MS analyses were done on the final diets to determine the actual content of both DON and FB, as well as the natural presence of other major mycotoxins (conducted by Romer Labs, Union, MO, USA; aflatoxin B_1_ found in the final diets from 2.9 to 9.8 μg/kg of feed). Diet analyses, including the ingredient formulation and nutrient composition, are reported in Table 5.

##### Birds and Housing

**Figure 6:**
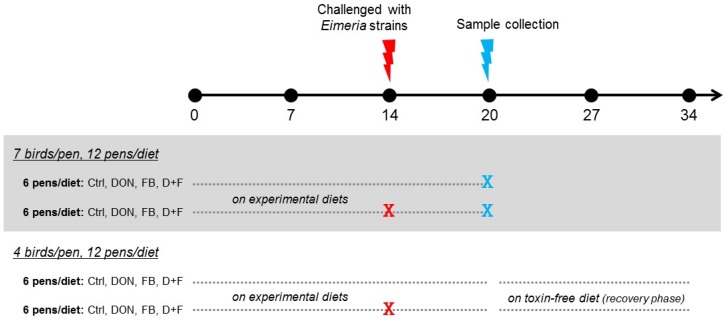
Experimental design: 336 birds (7 birds/pen, 12 pens/diet) were fed the experimental diets until day 20, half were orally challenged at day 14 with 25× of coccidial vaccine, and all were sampled at day 20. In parallel, 192 birds (4 birds/pen, 12 pens/diet) were fed the same experimental diets until day 20, half were orally challenged at d 14 with 25× of coccidial vaccine, and at day 20 all of the birds were kept on basal feed until day 34.

In total, 528 birds were used for this study. All of the birds were allocated to the experimental diets during the first phase (day 0–20). However, 336 birds were allocated as twelve replicate pens with 7 birds per pen, and 192 birds allocated as twelve replicate pens with 4 birds per pen (Figure 6). The latter were raised in the same conditions, having access to the same experimental diets, but were not sampled at day 20. By contrast, they were all kept on toxin free-diet for an additional 2 weeks, which was considered as the recovery phase (Figure 6). Birds were housed in stainless-steel battery brooders equipped with nipple-type waterers and thermostatically controlled heaters. The mortality of birds was recorded daily.

##### Challenge

On day 14, half of the birds (264 birds) were challenged with an overdose (25 times manufacturer recommendation) of coccidial vaccine (Coccivac-B, Schering-Plough Animal Health Corp., Millsboro, DE, USA) by oral gavage (Figure 6). The vaccine is a live oocyst vaccine, prepared from anticoccidial-sensitive strains of *Eimeria acervulina*, *E. mivati*, *E. maxima* and *E. tenella*. One dose of vaccine (the dose recommended by the manufacturer) contained approximately 500 oocysts based on the use of McMaster method [43] to enumerate visible oocysts. Accordingly, each bird in the present experiment received approximately 12,500 oocysts.

##### Sampling

Body weight and feed intake were measured on 0, 7, 14, 20, 27 and 34 days of age. On day 20, the 336 birds (challenged and unchallenged) not intended for the recovery phase were euthanized by an overdose of carbon dioxide. The digesta contents from the entire ileum (section between Meckel’s diverticulum and about 2 cm anterior to the ileo-cecal-colonic junction) were flushed with water into a clean plastic container, pooled within pen (7 birds/pen), and stored at −20 °C until it was freeze-dried. Duodenum, mid-jejunum, and cecum were observed and scored for gross lesions typically induced by the *Eimeria* strains used (12 birds per diet). The mucosa of the mid-jejunum from six birds/diet (1 bird/pen, 6 pens/diet; average weight) was scraped for the determination of oocyst number. Oocyst enumeration was also conducted on pools of excreta harvested at day 20 (1 pool/pen, 6 pens/diet). From six other birds/diet (1 bird/pen, 6 pens/diet; average weight), a portion of mid-jejunum was also taken, placed in cryovials containing RNAlater (Ambion Inc., Austin, TX, USA) for subsequent RNA isolation, and also another portion fixed in 10% neutral-buffered formalin (Sigma Aldrich, Saint Louis, MO, USA) for histological analysis. From the same six birds/diet, blood was collected and centrifuged for serum collection (1000 g, 20 min, and 4 °C), liver and ileum were harvested, flash frozen in liquid nitrogen and stored at −80 °C, for future sphingolipid analyses.

#### 5.1.2. Experimental Parameter Measures

##### Digestibility and Retention Analysis

Feed, excreta, and ileal digesta were analyzed for determination of nutrient digestibility and retention. Dry matter content was determined on ground diets and freeze-dried ileal digesta and excreta by drying the samples at 100 °C for 24 h. Chromium was determined by the inductively coupled plasma atomic emission spectroscopy method (method 990.08; AOAC International, 2000) following nitric-perchloric acid wet ash digestion. Gross energy determinations of feed and excreta samples were performed in a Parr adiabatic bomb calorimeter (Parr Insturments Co., Moline, IL, USA) with benzoic acid as a standard. Nitrogen content of feed, ileal digesta, and excreta samples was determined using a Leco model FP 2000 N combustion analyzer (Leco Corp., St. Joseph, MI, USA). The apparent digestibility (ileal digesta) and retention (excreta) of DM and N were determined using the index method (Cr as the digestive marker) by adapting the formula of Hill and Anderson [44] as described by NRC [45].

##### Lesion Score and Oocyst Counting

Given the strains present in the live vaccine target specifically different sections of the GIT, the duodenum, the mid-jejunum, and the cecum were examined in a blind-way for gross lesions and scored according to the method of Johnson and Reid [46] and Conway and McKenzie [47]. Oocyst counting was done in both jejunal mucosa and excreta. In short, samples kept at 4 °C were weighed (≈500 mg), passed and stirred through a sieve with 300 mL or 20 mL of saturated NaCl solution for excreta or mucosa, respectively. A sample of the mixture was transferred into the two chambers of a McMaster slide (FEC source, Banks, OR, USA), and after 5 min, the oocysts floating were counted under microscope at 10× magnification. The number of oocysts was expressed as oocyst per gram of feces or mucosa.

##### Sphingolipid Analysis

Determination of So and Sa in serum and tissue samples, was performed as described in Grenier et al. [9]. In brief, 200 μL of serum were mixed with 0.6 mL of methanol/ACN (50/50, *v*/*v*), and shaken at room temperature for 30 min. Samples were centrifuged, the pellets were re-extracted with 0.3 mL of methanol/water (80/20, *v*/*v*) and centrifuged again. The combined supernatants were dried under compressed air and reconstituted in 600 μL of methanol/water (80/20, *v*/*v*). HPLC-MS/MS analysis was conducted on an Agilent 1290 series UHPLC system coupled to a 5500 Triple Quad mass spectrometer (Sciex, Framingham, MA, USA). Chromatographic separation was achieved on a C18 Gemini column (150 × 4.6 mm, 5 μm, Phenomenex, Torrance, CA, USA) at 30 °C and a flow rate of 0.9 mL/min, injection volume was 1 μL. A gradient elution program was performed with methanol/water/formic acid (40/59.85/0.15, *v*/*v*/*v*) and methanol/formic acid (99.85/0.15, *v*/*v*) as mobile phases. Tandem MS detection was carried out in positive ion mode after electrospray ionization at 550 °C (ion spray voltage: 5500 V). The declustering potential (DP), collision energy (CE) and Q1 and Q3 m/z for quantifier and qualifier transitions were for So DP 71 V, 300.3→282.3 (CE 15 eV), and 300.3→252.2 (CE 23 eV), respectively, and for Sa 146 V, 302.3→284.4 (CE 19 eV), and 302.3→60.1 (CE 21 eV), respectively. Samples of liver and ileum were homogenized and extracted as described in Grenier et al. [9], except that liver samples were diluted 7.5-fold and ileum samples were diluted 6-fold with methanol/water (80/20, *v*/*v*) before injection (1 µL). The same instruments, settings and eluents as described above were used, however, the chromatographic column was a Kinetex C18 (150 × 2.1 mm, 2.6 µm) fitted with a UHPLC C18 SecurityGuard ULTRA Cartridge (both Phenomenex, Torrance, CA, USA). The flow rate was set to 250 µL/min and the proportion of B was increased linearly from 35% to 100% (reached at 6.5 min). After a hold-time of 3.5 min at 100% B, the column was re-equilibrated for 2.4 min at 35% B. The same transitions as described for serum were used, however, quantifications are based on 300.3→252.2 for So and 302.3→60.1 for Sa. Apparent recoveries were determined by spiking the different matrices with six different levels of Sa and So (in triplicates) before sample preparation (serum: Sa 94%, So 91%; liver: Sa 59%, So 64%, ileum: Sa 80%, So 83%). Analyst software version 1.6.2 was used for instrument control, data evaluation was performed using Multiquant 3.0 software (both Sciex, Framingham, MA, USA). Concentrations of So and Sa were determined on the basis of external standard calibration functions (So and Sa standards purchased from Avanti Polar Lipids, Inc., Alabaster, Alabama, AL, USA).

##### Gene Expression Analysis

Jejunal tissue was processed in lysing bead tubes containing guanidine-thiocyanate acid phenol (QIAzol reagent, Qiagen, Valencia, CA, USA) for use with the FastPrep-24 (MP Biomedicals). Concentrations, integrity and quality of RNA were determined spectrophotometrically using Nanodrop ND1000 (Fisher Scientific, St. Louis, MO, USA). Two micrograms of total RNA were treated with DNase I (Sigma Aldrich, Saint Louis, MO, USA), and then reverse-transcribed using M-MLV reverse transcriptase (Promega, Madison, WI, USA). Real-time PCR was performed using iCycler iQ real-time PCR detection system (Bio-Rad, Hercules, CA, USA) with the iQ SYBR Green Supermix (Bio-Rad). Thermal cycling conditions for the PCR reactions were 95 °C for 5 min followed by 40 cycles of 95 °C for 10 s, then 55 °C for 20 s, and finishing at 72 °C for 20 s. RNA non-reverse transcript was used as the non-template control for verification of a no genomic DNA amplification signal. Each sample was assessed in duplicate on two separate plates (in triplicate if high coefficient of variation). Specificity of PCR products was checked at the end of the reaction by analyzing the curve of dissociation. In addition, the size of amplicons was verified by electrophoresis. The sequences of the primers used are detailed in Table 6. Amplification efficiency and initial fluorescence were determined by DART-PCR method [48]. Then, values obtained for each gene were normalized by both housekeeping genes glyceraldehyde 3-phosphate dehydrogenase (GAPDH) and ribosomal protein L4 (RPL4). Finally, gene expression was expressed relative to the control group.

##### Histological Analysis

After routine processing, sections were embedded in paraffin, the jejunal samples were sectioned at 2–4 μm slides parallel to the villi axis and stained by haematoxylin and eosin using standard procedures. The resulting slides were viewed at 10× magnification, 3 sections per slide (1 slide/bird) were observed, and 5 villi were counted per section, resulting in 15 measurements per slide.

#### 5.1.3. Statistics

One way ANOVA (IBM SPSS Version 19.0, IBM corp., New York, NY, USA, 2010) was first used to examine the effects of diets on the dependent variables (unchallenged and challenged groups analyzed separately). When main effects were significant (*p* < 0.05), differences between means were analyzed by the Fisher’s Least Significance Difference test or Games–Howell test in case the equal variances were not assumed (Levene statistic).

To determine the type of interaction between DON and FB, a two-way factorial ANOVA (IBM SPSS Version 19.0, IBM corp., New York, NY, USA, 2010) was applied with DON and FB as two different factors, and the presence or absence of mycotoxins is said to be a level of the factor (0 or 1.5 mg/kg for DON, and 0 or 20 mg/kg for FB). An interaction means that the effect of one factor depends on the level of another factor. If the interaction is significant (*p* < 0.05), it could be due to synergism or interference. When two factors act synergistically, the combined effect is greater than the sum of the separate effects. In contrast, when two factors in combination inhibit each other’s effects it is termed interference or antagonism. If the interaction is not significant (*p* > 0.05), the combined effect is equal to the sum of the separate effects and termed additive. 

The analysis studying the relationship between ∆BWG and ∆FI (as shown in Figure 1) used linear regressions: ∆BWG = α + β × ∆FI, based on Pastorelli et al. [24]. The intercept (α) reflects the reduction in BWG not related to the reduction in FI, which can be interpreted as an indicator for maintenance. The slope (β) reflects the extent of the change in BWG associated with the reduction in FI between challenged and unchallenged birds, and is an indicator of the feed efficiency. The Pearson correlation coefficient was determined as a measure of the linear correlation between body weight gain and feed intake (d14–20).

### 5.2. Experiment 2: Feeding Trial with Diets Contaminated with DON

#### Experimental Birds, Diet Formulation, Sampling and Experimental Parameter Measures

The exact same experimental timeline and housing were used in Experiment 2. Briefly, 32 male broilers were allocated to two experimental diets (16 birds/diet, 2 pens/diet, 8 birds/pen), control and DON diets. The same culture material as in Experiment 1 was used to formulate the DON diet. The actual content of DON was determined by HPLC-MS analysis (conducted by Romer Labs, Union, MO, USA), and DON was found at 7.6 mg/kg of feed. Aflatoxins and fumonisins were below the limit of detection. On d 14, half of the birds (16 birds) were challenged with an overdose (25×) of the coccidial vaccine by oral gavage. On d 20, the 32 birds (challenged and unchallenged) were euthanized as previously described. Cecal tonsils were collected for flow cytometry and gene expression analyses. The procedure for determination of mRNA levels is exactly the same as in Experiment 1. Regarding flow cytometry analysis, single cell suspensions of one cecal tonsil was concentrated for mononuclear cells by density centrifugation at 400× *g* for 20 min over Histopaque (1.077 g/mL; Sigma Aldrich, Saint Louis, MO, USA) and incubated with 10 μg/mL of PE-conjugated mouse anti-chicken CD25 (kindly provided by Dr R.K. Selvaraj, Department of Animal Sciences, Wooster, OH, USA; and produced as described earlier [23]), 1:200 FITC-conjugated mouse anti-chicken CD4 (Southern Biotech, Birmingham, AL, USA), or 1:200 PE-conjugated mouse anti-chicken CD8 (Southern Biotech, Birmingham, AL, USA) for 60 min. The unbound antibodies were removed by centrifugation at 750× *g* for 10 min. The percentages of CD4^+^, CD8^+^, and CD4^+^CD25^+^ cells (T_regs_) in cecal tonsil were analyzed by flow cytometry (BD Accuri C6 Cytometer, San Jose, CA, USA) after gating the area corresponding to mononuclear cells. The CD4^+^ and CD8^+^ cells were expressed as a percentage of mononuclear cells. The percentage of T_regs_ was expressed as a percentage of CD4^+^ cells to facilitate comparison between samples.

## Figures and Tables

**Table 1:** Body weight gain of challenged and unchallenged birds fed experimental diets, and of birds fed control feed during the recovery phase (Experiment 1).

BWG per Bird (g)	Unchallenged				Challenged			
	Ctrl	DON	FB	DON + FB	Ctrl	DON	FB	DON + FB
7 birds/pen								
d0–d14	334 ± 7 ^a,b^	357 ± 10 ^b^	343 ± 11 ^b^	303 ± 12 ^a^	334 ± 13	354 ± 14	340 ± 8	332 ± 8
d14–d20	300 ± 20	347 ± 12	325 ± 6	312 ± 10	225 ± 15	207 ± 6	225 ± 8	202 ± 15
4 birds/pen								
d0–d14	337± 12	342 ± 13	352 ± 13	330 ± 20	301 ± 12 ^a^	379 ± 12 ^b^	337 ± 11 ^a,b^	333 ± 11 ^a^
d14–d20	339 ± 18	332 ± 17	331 ± 15	322 ± 27	195 ± 10 ^a,b^	231 ± 12 ^b^	188 ± 12 ^a^	200 ± 15 ^a,b^
d20–d34 *	1014 ± 31	997 ± 46	984 ± 69	962 ± 62	981 ± 9	1022 ± 31	986 ± 21	1021 ± 12

Results are expressed for six replicates ± SEM. Means within a row (unchallenged and challenged groups analyzed separately) with no common superscript are significantly different (*p <* 0.05) for this time point. *, day 20 to 34: recovery phase with all birds kept on toxin-free diet. BWG, Body Weight Gain; DON, Deoxynivalenol; FB, Fumonisins.

**Table 2:** Effects of the contaminated diets on morphometry in the jejunum and on apparent dry matter and nitrogen digestibility of unchallenged and challenged chickens (Experiment 1).

Groups	Apparent DM Digestibility (%)	Apparent N Digestibility (%)	Villus Height (μm/villus)
**Unchallenged birds**			
Control	63.7 ± 0.9	79.6 ± 0.9	862 ± 31
DON	67.1 ± 1.4	81.8 ± 0.7	892 ± 42
FB	63.5 ± 0.9	80.2 ± 0.5	829 ± 24
DON + FB	62.6 ± 1.6	80.5 ± 0.8	802 ± 27
**Challenged birds**			
Control	32.7 ± 4.8	56.6 ± 2.1 ^a^	ND
DON	36.6 ± 5.4	56.3 ± 3.3 ^a,b^	ND
FB	42.2 ± 4.7	60.9 ± 2.5 ^a^	ND
DON + FB	32.1 ± 4.4	48.8 ± 3.2 ^b^	ND

Results are expressed for 6 pools (digestibility) or 6 birds (villus) ± SEM. Means within a column (unchallenged and challenged groups analyzed separately) with no common superscript are significantly different (*p <* 0.05). ND: No Data (destruction of the intestinal epithelium).

**Table 3:** Interaction between DON and FB in challenged birds (type of interaction between DON and FB determined by two-way factorial ANOVA when at least one of the contaminated diet (DON, FB, or DON + FB) was significantly different from the control diet for the endpoint assessed; Experiment 1).

Markers Assessed	No Effect of DON, FB or DON + FB —No Interaction Determined *	Type of Interaction between DON and FB *		
		Antagonistic	Additive	Synergistic
markers of pathogenicity	-	Lesions jejunum (0.036)	Oocyst excreta (0.47)	-
	-	-	Oocyst mucosa (0.21)	-
	-	-	Lesions duod. (0.17)	-
	-	-	Lesions ceca (0.47)	-
markers of gut inflammation	IL-17	IL-6 (0.036)	IL-8 (0.14)	-
	IL-21	IL-1β (0.095, tendency)	IL-10 (0.19)	-
	IFN-γ		SOCS1 (0.47)	-
markers of gut integrity	MUC2	-	SGLT1 (0.71)	Digestibility N (0.047)
	Digestibility DM	-	-	-
marker of exposure/effect	Sa/So ileum	-	Sa/So liver (0.47)	Sa/So serum (0.007, potentiation)
number of endpoints	6	3	9	2
relative to total endpoints	30%	15%	45%	10%

* The separate effects of DON, FB, or DON + FB versus control were first analyzed in one-way ANOVA followed by post-hoc test. When at least one of the contaminated diets were found significantly different with the control diet, then the two-way factorial ANOVA was carried out on that endpoint to determine the type of interaction between DON and FB (synergistic, additive, or antagonistic). The endpoints that were not significantly affected by the feeding of mycotoxin contaminated diets are reported in the column “no effect of DON, FB, or DON + FB—no interaction determined”. The next three columns report the output of the two-way factorial analysis with the *p* value in brackets for each endpoint. When *p* < 0.05, this suggests a synergistic or antagonistic interaction between DON and FB on the endpoint assessed. When two factors act synergistically, the combined effect is greater than the sum of the individual effects. In contrast, when two factors in combination inhibit each other’s effects it is termed antagonism. When *p* > 0.05, the interaction is then considered additive, the combined effect of DON and FB is equal to the sum of the individual effects of DON or FB when fed alone.

**Table 4:** Lymphocyte sub-population and gene expression in the cecal tonsils of unchallenged and challenged chickens fed DON (Experiment 2).

Parameters	Unchallenged		Challenged	
	Control	DON	Control	DON
Lymphocyte (%)				
CD4^+^CD25^+^	12 ± 3 ^a^	12 ± 4 ^a^	27 ± 3 ^b^	45 ± 7 ^c^
CD4^+^	45 ± 3 ^a^	42 ± 2 ^a^	52 ± 2 ^a,b^	66 ± 4 ^b^
CD8^+^	36 ± 2 ^a^	35 ± 5 ^a,b^	25 ± 3 ^b^	40 ± 5 ^a^
Gene expression				
IL-1β	1.00 ± 0.14 ^a^	1.01 ± 0.14 ^a^	1.79 ± 0.53 ^a,b^	3.19 ± 0.68 ^b^
IL-6	1.00 ± 0.09 ^a^	0.59 ± 0.08 ^b^	4.73 ± 1.54 ^c^	4.57 ± 0.84 ^c^
IL-17	1.00 ± 0.10 ^a^	0.85 ± 0.10 ^a^	0.56 ± 0.06 ^b^	0.50 ± 0.07 ^b^
IFN-γ	1.00 ± 0.11 ^a^	1.02 ± 0.16 ^a^	4.41 ± 0.65 ^b^	8.49 ± 1.41 ^c^
IL-10	1.00 ± 0.16 ^a^	1.08 ± 0.20 ^a^	1.97 ± 0.36 ^b^	2.27 ± 0.22 ^b^
SOCS1	1.00 ± 0.07	0.98 ± 0.15	1.32 ± 0.08	1.38 ± 0.19

Results are expressed for 5 birds (lymphocyte) and 8 birds (gene expression) ± SEM. Means within a row (unchallenged and challenged groups analyzed together) with no common superscript are significantly different (*p <* 0.05).

**Table 5:** Diet formulation, nutrient specification and mycotoxin content (Experiment 1).

Item	Starter Diet (day 0–20)	Grower Diet (day 21–34)
Ingredient (% of diet)		
Corn	53.98	52.10
Soybean meal (48% CP)	38.05	39.09
Soy oil	3.52	5.00
Sodium chloride	0.48	0.46
DL-Methionine	0.25	0.24
Threonine	0.07	-
L-Lysine, HCl	0.10	-
Limestone	1.68	1.56
Monocalcium phosphate	1.52	1.20
Vitamin and mineral premix ^1^	0.35	0.35
Nutrient composition (calculated)		
ME, kcal/kg	3066	3151
CP, %	22.43	22.81
Ca, %	1.01	0.85
Non-phytate phosphorus, %	0.43	0.44
Met, %	0.59	0.59
Thr, %	0.92	0.89
Lys, %	1.34	1.29
Analyzed composition	Deoxynivalenol (mg/kg)	Fumonisins ^2^ (mg/kg)
Control diet	-	3.1
DON diet	1.6	2.9
FB diet	-	20.5
DON + FB diet	1.3	20.8

^1^ Supplied per kilogram of diet: vitamin A, 13,233 IU; vitamin D3, 6636 IU ; vitamin E, 44.1 IU ; vitamin K, 4.5 mg; thiamine, 2.21 mg; riboflavin, 6.6 mg; pantothenic acid, 24.3 mg; niacin, 88.2 mg; pyridoxine, 3.31 mg; folic acid, 1.10 mg; biotin, 0.33 mg; vitamin B12, 24.8 μg; choline, 669.8 mg; iron from ferrous sulfate, 50.1 mg; copper from copper sulfate, 7.7 mg; manganese from manganese oxide, 125.1 mg; zinc from zinc oxide, 125.1 mg; iodine from ethylene diamine dihydroidide, 2.10 mg; selenium from sodium selenite, 0.25 mg; ^2^ Total fumonisins as the sum of fumonisin B_1_ + B_2_ + B_3_.

**Table 6:** Nucleotide sequence of primers for real-time PCR.

Gene	Primer Sequence	Amplicon		Ensembl Access	Reference
		Size	Intron ^1^		
Housekeeping genes					
GAPDH	F (300 nM) TCCTAGGATACACAGAGGACCA R (300 nM) CGGTTGCTATATCCAAACTCA	151 bp	2 (499)	ENSGALG00000014442	[9]
RPL4	F (300 nM) TTATGCCATCTGTTCTGCC R (300 nM) GCGATTCCTCATCTTACCCT	235 bp	2 (893)	ENSGALG00000007711	[9]
Pro-inflammatory cytokines					
IL-1β	F (300 nM) GCATCAAGGGCTACAAGCTC R (300 nM) CAGGCGGTAGAAGATGAAGC	131 bp	1 (87)	ENSGALG00000000534	[22]
IL-6	F (300 nM) GAATGTTTTAGTTCGGGCACA R (300 nM) TTCCTAGAAGGAAATGAGAATGC	130 bp	0	ENSGALG00000010915	[9]
IL-8	F (300 nM) GCGGCCCCCACTGCAAGAAT R (300 nM) TCACAGTGGTGCATCAGAATTGAGC	146 bp	2 (1210)	ENSGALG00000011670	[9]
Treg signature					
IL-10	F (300 nM) GCTGAGGGTGAAGTTTGAGG R (300 nM) AGACTGGCAGCCAAAGGTC	121 bp	2 (1127)	ENSGALG00000000892	[9]
SOCS1	F (300 nM) CAAGCGGATTTCAGTAGCATC R (300 nM) GGCTCAGACTTCAGCTTCTCA	110 bp	no intron	ENSGALG00000007158	[9]
Th17 & Th1 signature					
IL-17	F (300 nM) TATCAGCAAACGCTCACTGG R (300 nM) AGTTCACGCACCTGGAATG	110 bp	1 (666)	ENSGALG00000016678	[9]
IL-21	F (300 nM) GCTTTCAAAGACAATTGACCATC R (300 nM) TACAGCTGTGAGCAGGCATC	106 bp	2 (3765)	ENSGALG00000011844	[9]
IFN-γ	F (300 nM) AGCTGACGGTGGACCTATTATT R (300 nM) GGCTTTGCGCTGGATTC	259 bp	2 (998)	ENSGALG00000009903	[37]
Gut integrity					
SGLT1	F (300 nM) CATCTTCCGAGATGCTGTCA R (300 nM) AGGTATCCGCACATCACACA	168 bp	1 (979)	ENSGALG00000006728	Present study
MUC2	F (300 nM) CAGCACCAACTTCTCAGTTCC R (300 nM) TCTGCAGCCACACATTCTTT	102 bp	1 (1711)	ENSGALG00000006752	Present study

^1^ number of introns spanned in the design of primers, the brackets report the total size of introns (in bp). GAPDH, Glyceraldehyde 3-phosphate dehydrogenase; IFN-γ, Interferon-γ; IL, Interleukin; MUC2, Mucin 2; RPL4, Ribosomal protein L4; SGLT1, Sodium-dependent glucose cotransporter 1; SOCS1, Suppressor of cytokine signaling 1.

## Abbreviations

The following abbreviations are used in this manuscript:

DON	Deoxynivalenol
FB	Fumonisins
GIT	Gastrointestinal Tract
Sa	Sphinganine
So	Sphingosine

## References

[1] Oswald I.P., Marin D.E., Bouhet S., Pinton P., Taranu I., Accensi F. (2005). Immunotoxicological risk of mycotoxins for domestic animals. Food Addit. Contam..

[2] Bryden W.L. (2012). Mycotoxin contamination of the feed supply chain: Implications for animal productivity and feed security. Anim. Feed Sci. Technol..

[3] Grenier B., Applegate T. (2013). Modulation of intestinal functions following mycotoxin ingestion: Meta-analysis of published experiments in animals. Toxins.

[4] Streit E., Naehrer K., Rodrigues I., Schatzmayr G. (2013). Mycotoxin occurrence in feed and feed raw materials worldwide: Long-term analysis with special focus on europe and asia. J. Sci. Food Agric..

[5] Girgis G.N., Sharif S., Barta J.R., Boermans H.J., Smith T.K. (2008). Immunomodulatory effects of feed-borne fusarium mycotoxins in chickens infected with coccidia. Exp. Biol. Med..

[6] Kana J.R., Teguia A., Tchoumboue J. (2010). Effect of dietary plant charcoal from canarium schweinfurthii engl. And maize cob on aflatoxin b1 toxicosis in broiler chickens. Adv. Anim. Biosci..

[7] Awad W.A., Vahjen W., Aschenbach J.R., Zentek J. (2011). A diet naturally contaminated with the fusarium mycotoxin deoxynivalenol (don) downregulates gene expression of glucose transporters in the intestine of broiler chickens. Livest. Sci..

[8] Antonissen G., Van Immerseel F., Pasmans F., Ducatelle R., Haesebrouck F., Timbermont L., Verlinden M., Janssens G.P.J., Eeckhaut V., Eeckhout M. (2014). The mycotoxin deoxynivalenol predisposes for the development of clostridium perfringens-induced necrotic enteritis in broiler chickens. PLoS ONE.

[9] Grenier B., Schwartz-Zimmermann H., Caha S., Moll W., Schatzmayr G., Applegate T. (2015). Dose-dependent effects on sphingoid bases and cytokines in chickens fed diets prepared with fusarium verticillioides culture material containing fumonisins. Toxins.

[10] Antonissen G., Van Immerseel F., Pasmans F., Ducatelle R., Janssens G., De Baere S., Mountzouris K., Su S., Wong A., De Meulenaer B. (2015). Mycotoxins deoxynivalenol and fumonisins alter the extrinsic component of intestinal barrier in broiler chickens. J. Agric. Food Chem..

[11] Yunus A.W., Razzazi-Fazeli E., Bohm J. (2011). Aflatoxin b1 in affecting broiler’s performance, immunity, and gastrointestinal tract: A review of history and contemporary issues. Toxins.

[12] Cheema M., Qureshi M., Havenstein G. (2003). A comparison of the immune response of a 2001 commercial broiler with a 1957 randombred broiler strain when fed representative 1957 and 2001 broiler diets. Poult. Sci..

[13] Chapman H.D. (2014). Milestones in avian coccidiosis research: A review. Poult. Sci..

[14] Dalloul R.A., Lillehoj H.S. (2006). Poultry coccidiosis: Recent advancements in control measures and vaccine development. Expert Rev. Vaccines.

[15] Li G.Q., Kanu S., Xiao S.M., Xiang F.Y. (2005). Responses of chickens vaccinated with a live attenuated multi-valent ionophore-tolerant eimeria vaccine. Vet. Parasitol..

[16] Farnell M.B., Donoghue A.M., de los Santos F.S., Blore P.J., Hargis B.M., Tellez G., Donoghue D.J. (2006). Upregulation of oxidative burst and degranulation in chicken heterophils stimulated with probiotic bacteria. Poult. Sci..

[17] Girgis G.N., Barta J.R., Brash M., Smith T.K. (2010). Morphologic changes in the intestine of broiler breeder pullets fed diets naturally contaminated with fusarium mycotoxins with or without coccidial challenge. Avian Dis..

[18] Girgis G.N., Barta J.R., Girish C.K., Karrow N.A., Boermans H.J., Smith T.K. (2010). Effects of feed-borne fusarium mycotoxins and an organic mycotoxin adsorbent on immune cell dynamics in the jejunum of chickens infected with eimeria maxima. Vet. Immunol. Immunopathol..

[19] Grenier B., Oswald I. (2011). Mycotoxin co-contamination of food and feed: Meta-analysis of publications describing toxicological interactions. World Mycotoxin J..

[20] Chou T.-C. (2006). Theoretical basis, experimental design, and computerized simulation of synergism and antagonism in drug combination studies. Pharmacol. Rev..

[21] Alassane-Kpembi I., Kolf-Clauw M., Gauthier T., Abrami R., Abiola F.A., Oswald I.P., Puel O. (2013). New insights into mycotoxin mixtures: The toxicity of low doses of type b trichothecenes on intestinal epithelial cells is synergistic. Toxicol. Appl. Pharmacol..

[22] Adedokun S.A., Ajuwon K.M., Romero L.F., Adeola O. (2012). Ileal endogenous amino acid losses: Response of broiler chickens to fiber and mild coccidial vaccine challenge. Poult. Sci..

[23] Shanmugasundaram R., Selvaraj R.K. (2011). Regulatory t cell properties of chicken CD4^+^CD25^+^ cells. J. Immunol..

[24] Pastorelli H., van Milgen J., Lovatto P., Montagne L. (2012). Meta-analysis of feed intake and growth responses of growing pigs after a sanitary challenge. Animal.

[25] Voss K.A., Smith G.W., Haschek W.M. (2007). Fumonisins: Toxicokinetics, mechanism of action and toxicity. Anim. Feed Sci. Technol..

[26] Lillehoj H.S., Min W., Dalloul R.A. (2004). Recent progress on the cytokine regulation of intestinal immune responses to eimeria. Poult. Sci..

[27] Murugesan G.R., Ledoux D.R., Naehrer K., Berthiller F., Applegate T.J., Grenier B., Phillips T.D., Schatzmayr G. (2015). Prevalence and effects of mycotoxins on poultry health and performance, and recent development in mycotoxin counteracting strategies. Poult. Sci..

[28] Henry M.H., Wyatt R.D., Fletchert O.J. (2000). The toxicity of purified fumonisin b1 in broiler chicks. Poult. Sci..

[29] Eriksen G.S., Pettersson H. (2004). Toxicological evaluation of trichothecenes in animal feed. Anim. Feed Sci. Technol..

[30] Broomhead J.N., Ledoux D.R., Bermudez A.J., Rottinghaus G.E. (2002). Chronic effects of fumonisin b1 in broilers and turkeys fed dietary treatments to market age. Poult. Sci..

[31] Yunus A.W., Ghareeb K., Twaruzek M., Grajewski J., Böhm J. (2012). Deoxynivalenol as a contaminant of broiler feed: Effects on bird performance and response to common vaccines. Poult. Sci..

[32] Pestka J. (2010). Deoxynivalenol: Mechanisms of action, human exposure, and toxicological relevance. Arch. Toxicol..

[33] Stringfellow K., Caldwell D., Lee J., Mohnl M., Beltran R., Schatzmayr G., Fitz-Coy S., Broussard C., Farnell M. (2011). Evaluation of probiotic administration on the immune response of coccidiosis-vaccinated broilers. Poult. Sci..

[34] Paris N.E., Wong E.A. (2013). Expression of digestive enzymes and nutrient transporters in the intestine of eimeria maxima-infected chickens. Poult. Sci..

[35] Antonissen G., Devreese M., Van Immerseel F., De Baere S., Hessenberger S., Martel A., Croubels S. (2015). Chronic exposure to deoxynivalenol has no influence on the oral bioavailability of fumonisin b1 in broiler chickens. Toxins.

[36] Yun C.H., Lillehoj H.S., Lillehoj E.P. (2000). Intestinal immune responses to coccidiosis. Dev. Comp. Immunol..

[37] Hong Y.H., Lillehoj H.S., Lillehoj E.P., Lee S.H. (2006). Changes in immune-related gene expression and intestinal lymphocyte subpopulations following eimeria maxima infection of chickens. Vet. Immunol. Immunopathol..

[38] Jiang Z., Applegate T.J., Lossie A.C. (2013). Cloning, annotation and developmental expression of the chicken intestinal muc2 gene. PLoS ONE.

[39] Shanmugasundaram R., Selvaraj R.K. (2012). CD4^+^CD25^+^ regulatory t cell ontogeny and preferential migration to the cecal tonsils in chickens. PLoS ONE.

[40] Kubena L., Edrington T., Harvey R., Buckley S., Phillips T., Rottinghaus G., Casper H. (1997). Individual and combined effects of fumonisin b1 present in fusarium moniliforme culture material and t-2 toxin or deoxynivalenol in broiler chicks. Poult. Sci..

[41] Desjardins A.E., Plattner R.D., Shackelford D.D., Leslie J.F., Nelson P.E. (1992). Heritability of fumonisin b1 production in gibberella fujikuroi mating population a. Appl. Environ. Microbiol..

[42] Altpeter F., Posselt U.K. (1994). Production of high quantities of 3-acetyldeoxynivalenol and deoxynivalenol. Appl. Microbiol. Biotechnol..

[43] Hodgson J.N. (1970). Coccidiosis: Oocyst counting technique for coccidiostat evaluation. Exp. Parasitol..

[44] Hill F.W., Anderson D.L. (1958). Comparison of metabolizable energy and productive energy determinations with growing chicks. J. Nutr..

[45] NRC (1994). Nutrient Requirements of Poultry.

[46] Johnson J., Reid W.M. (1970). Anticoccidial drugs: Lesion scoring techniques in battery and floor-pen experiments with chickens. Exp. Parasitol..

[47] Conway D.P., McKenzie M.E. (2008). Examination of lesions and lesion scoring. Poultry Coccidiosis.

[48] Peirson S.N., Butler J.N., Foster R.G. (2003). Experimental validation of novel and conventional approaches to quantitative real-time pcr data analysis. Nucleic Acids Res..

